# Apixaban in a porcine model of mechanical valve thrombosis in pulmonary position—a pilot study

**DOI:** 10.1093/icvts/ivac070

**Published:** 2022-03-07

**Authors:** Lucas Van Hoof, Isabelle Truyers, Hadewych Van Hauwermeiren, Bram Nachtergaele, Tom Langenaeken, Marc Jacquemin, Filip Rega, Peter Verhamme, Bart Meuris

**Affiliations:** 1 Department of Cardiac Surgery, University Hospitals Leuven, Leuven, Belgium; 2 Medanex Clinic, Diest, Belgium; 3 Department of Hemostasis in Laboratory Medicine, University Hospitals Leuven, Leuven, Belgium; 4 Department of Vascular Medicine and Hemostasis, University Hospitals Leuven, Leuven, Belgium

**Keywords:** Mechanical valve, Thrombosis, Apixaban, Pig, On-X

## Abstract

**OBJECTIVES:**

The newest mechanical valves have low thrombogenicity, making them candidates for anticoagulation with a direct oral anticoagulant. While these drugs hold great promise to replace warfarin, clinical trials have been disappointing so far. We aimed to evaluate apixaban in a porcine model of mechanical valve thrombosis with On-X^®^ (CryoLife) aortic valves implanted in pulmonary position.

**METHODS:**

On-X^®^ valves were implanted in pulmonary valve position in 9 Yucatan pigs. Animals received prophylactic enoxaparin 40 mg for 1 week. Pigs in the low-dose group received 5 mg apixaban twice daily for 10 weeks. The intermediary-dose group received 5 mg twice daily for 6 weeks and then 10 mg twice daily afterwards. The high-dose group received 15 mg twice daily for 10 weeks. After sacrifice, valves were macroscopically evaluated and thrombus weight was documented.

**RESULTS:**

The median weight of the 9 animals was 64.3 kg, range 52.5–70.9. In the low-dose group (2 animals), both valves showed manifest, chronic thrombosis with blocked hinges. In the intermediary-dose group, a normal functioning valve without thrombosis was seen in 2/4 animals. In the high-dose group (3 pigs), there was no valve thrombosis. No bleeding events occurred. In all animals, apixaban plasma levels were low compared to clinical target levels.

**CONCLUSIONS:**

The pulmonary position seems to be an aggressive model for mechanical valve thrombosis in pigs. Apixaban has the potential to prevent valve thrombosis, even in these thrombogenic conditions. Detailed pharmacokinetic studies are needed to determine the ideal apixaban dosage for future experiments and to enable extrapolation to the clinical situation.

**Figure:**
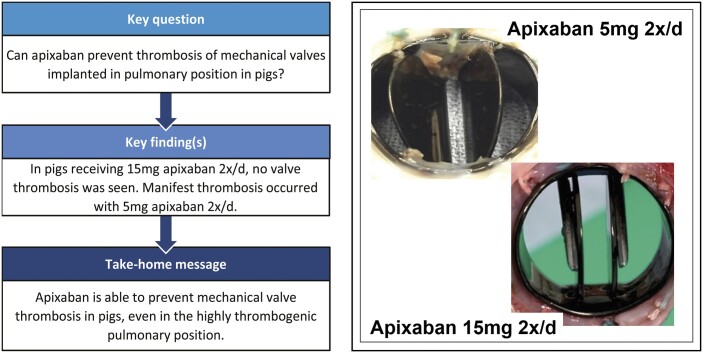

## INTRODUCTION

Mechanical valves currently require long-term anticoagulation with vitamin K antagonists. Their use remains problematic due to a narrow therapeutic range and bleeding- or thrombo-embolic complications, especially when international normalized ratio (INR) levels are out of range [1]. For venous thrombo-embolism and atrial fibrillation, direct oral anticoagulant (DOAC) drugs have a proven higher efficacy compared to warfarin with less major bleeding and without the need for chronic INR monitoring due to a predictable therapeutic profile [2, 3].

Due to their design and material properties, the newest generation mechanical valves (On-X^®^, CryoLife and Bicarbon^®^, LivaNova) have a proven safety and efficacy at an INR of 1.5–2.0 for aortic position instead of the typical 2.0–3.0, making them candidates to permit anticoagulation with a DOAC [4, 5]. A previous clinical trial evaluating dabigatran for mechanical valves was, however, terminated prematurely due to excess thrombo-embolism and bleeding [6]. This failure was probably due to a combination of patient selection, inadequate dosage, timing of initiation and the narrow working spectrum of dabigatran, preventing only thrombin formation. Anti-Xa inhibitors such as apixaban or rivaroxaban seem to be better candidates to replace warfarin due to their earlier and more powerful mechanism of action on the coagulation cascade [7–10]. The PROACT Xa trial evaluating apixaban compared to warfarin, both without the mandatory association of aspirin, in patients with an On-X^®^ aortic valve, is currently ongoing [11].

While sheep are often considered the gold standard for the preclinical evaluation of heart valve prostheses, there are concerns about insufficient thrombogenicity to evaluate mechanical valves in aortic position. Most notorious is the discontinuation of the Medtronic Parallel^®^ valve after an unusually high rate of valve thrombosis in patients, while this was not observed in sheep [12]. Pigs have been proposed as a more suitable model because, compared to sheep or cows, their coagulation system is most similar to that of humans [13–15]. Furthermore, pigs seem to be hypercoagulable compared to humans [15]. On the other hand, the reports of mechanical valve implantations in aortic or mitral position in pigs are scarce, with inconsistent thrombosis rates and a significant operative risk [16–18].

In a simplified porcine model with mechanical valved conduits bypassing the descending aorta, a high dosage of apixaban, dabigatran, melagatran or rivaroxaban drastically reduced valve thrombosis [8, 9, 19, 20]. This heterotopic model of aortic valve replacement has a low operative risk yet is likely excessively thrombogenic due to non-physiological blood flow and the absence of normal bileaflet cusp motion. Therefore, a clinically representative animal model remains needed to study the mechanisms of valve thrombosis and novel therapeutic strategies.

We have previously implanted mechanical valves in pulmonary position in 12 sheep, and thrombotic changes were seen in all valves [21]. Based on a literature study and on the previous work [21], we hypothesize that pulmonary position in pigs provides an aggressive model for mechanical valve thrombosis and that apixaban is able to prevent thrombosis. In this study, we aimed to evaluate the effect of orally administered apixaban in a porcine model of mechanical valve thrombosis with On-X^®^ (CryoLife, USA) aortic valves implanted in orthotopic pulmonary valve position.

## MATERIALS AND METHODS

### Ethics statement

This study was approved by the Medanex Clinic Ethical Committee (EC MxCl 2018–095). Animals were cared for in the Medanex Clinic (Webbekom, Belgium) under supervision by a veterinarian in accordance with the ‘Guide for the care and use of laboratory animals, 8th edition’, formulated by the National Research Council [22].

### Study design

Nine Yucatan pigs (median weight 64.3 kg, interquartile range 54.5–67.6, range 52.5–70.9) were acquired from the INRA (Institute National de La Recherche Agronomique, France). A 21-mm On-X^®^ aortic valve was implanted in all animals.

### Surgery

Prior to surgery, animals were fasted for 24 h. Pigs were sedated using intramuscular midazolam and xylazine. An arterial pressure line and venous catheter were inserted in the ear. Anaesthesia was induced and maintained using intravenous propofol. After intubation, mechanical ventilation was started. Ceftiofur was administered as antibiotic prophylaxis. Animals were positioned in a right lateral recumbent position, surgically scrubbed and draped to expose the left chest wall.

A left anterolateral thoracotomy was performed in the third intercostal space. Lidocaine was given to prevent ventricular arrhythmias. The pericardium was incised in a T-shaped fashion directly over the main pulmonary artery and the heart was suspended in a pericardial cradle. After dissection of the aorto-pulmonary window and heparinization (300 IU/kg, target activating clotting time (ACT) > 500), normothermic cardiopulmonary bypass was established by cannulating the ascending aorta and right atrial appendage. Once sufficient venous drainage was confirmed, the distal pulmonary artery was clamped. The pulmonary artery was opened anteriorly at the level of the sinotubular junction. A suction catheter in the right ventricle (via the incised pulmonary artery) enabled a dry working field. A 21-mm On-X^®^ aortic valve was implanted in orthotopic position after excising the native pulmonary valve leaflets. Position and function were verified thoroughly prior to de-airing and closure of the pulmonary artery. The pig was weaned from CPB and once haemodynamic parameters are stable, the heart-lung machine was stopped and cannulas removed. The pericardium was left open and the chest closed in a standard fashion over a drain.

### Postoperative care and medication regimen

Animals were extubated and transferred to the intensive care unit after confirming that they were awake, breathing spontaneously and haemodynamically stable with sufficient hemostasis. The chest drain was removed on clinical indication, usually after 1 h. In the first 3 postoperative days, meloxicam and buprenorphine are used for analgesia. Ceftiofur and enoxaparin (40 mg/day subcutaneously) are given the first 7 days while furosemide (1 mg/kg) is given as indicated. For this study, oral apixaban was started after 1 week and given for 10 weeks. The animal caretakers verified that the animals ingested each tablet, and general well-being and signs of bleeding were evaluated 3 times per day. Two animals (low-dose group) received 5 mg apixaban twice daily for 10 weeks. Four animals (intermediary-dose group) initially received 5 mg twice daily for 6 weeks. Upon observing manifest valve thrombosis in the low-dose group, the dosage was increased to 10 mg twice daily in these 4 animals for the remainder of the study (Table 1). Three animals (high-dose group) received 15 mg twice daily for 10 weeks.

**Table 1:** Overview of all animals including weight at operation, apixaban regimen, apixaban peak plasma concentration and thrombus weight on the explanted valve

Group	Animal nr	Weight	Apixaban regimen	Normalized apixaban dose (mg/kg/d)	Apixaban peak (ng/ml)	Thrombus weight (mg)
Low dose	1	64.3	2 × 5 mg 10 weeks	0.16	23.07	108.8
	2	66.1	2 × 5 mg 10 weeks	0.15	64.05	548.8
Intermediary dose	3	52.6	2 × 5 mg 6 weeks to 2 × 10 mg 2 weeks	0.24	–	65.5
	4	58.9	2 × 5 mg 6 weeks to 2 × 10 mg 4 weeks	0.24	39.88	41
	5	52.5	2 × 5 mg 6 weeks to 2 × 10 mg 4 weeks	0.27	60.99	0
	6	54.5	2 × 5 mg 6 weeks to 2 × 10 mg 4 weeks	0.26	49.91	0
High dose	7	70.1	2 × 15 mg 10 weeks	0.43	44.6	0
	8	67.6	2 × 15 mg 10 weeks	0.44	49.9	0
	9	70.9	2 × 15 mg 10 weeks	0.42	82.9	0

### Apixaban plasma concentrations

Venous blood samples in sodium citrate tubes were taken on the day of autopsy, 3 h after the last ingestion of apixaban to obtain peak plasma concentrations. Blood samples were centrifuged, and the plasma was removed and stored at −80°C until further processing. Apixaban anti-Xa assays were performed using an ACL TOP 500 CTS System (Werfen, USA). In brief, factor Xa was added to a mixture of undiluted plasma and chromogenic substrate S-2732. Apixaban in the plasma sample directly neutralizes factor Xa. Residual factor Xa reacts with the chromogenic substrate to form paranitroaniline. The paranitroaniline release, quantified via spectrophotometry at 405 nm, is inversely proportional to the apixaban concentration (ng/ml) in the sample. Prior to testing the samples, the assay was validated by comparing spiked human and porcine plasma.

### Explant procedures

At study completion, fluoroscopic X-ray evaluation of the valves was performed under sedation to evaluate leaflet mobility. Prior to euthanasia with pentobarbital, heparin (300 IU/kg) was given to prevent post-mortem thrombosis. A cardiectomy was performed, the lungs were macroscopically evaluated and random lung biopsies were taken to screen for thrombo-embolism. In case of visible lesions, these would be examined separately. A complete necropsy was performed to evaluate signs of bleeding. After carefully rinsing the explanted tissue, the valves were evaluated for the following parameters: thrombus, vegetation, tissue overgrowth, paravalvular leak and valve integrity. Macroscopic photographs were taken. If present, thrombus was removed, weighed and processed for histological evaluation.

### Data analysis

Continuous variables were reported as median (interquartile range when appropriate, range) and compared using the Mann–Whitney *U*-test or independent-samples Kruskal–Wallis test to evaluate the distribution of variables across treatment groups. The Bonferroni correction was used in case of multiple tests. Spearman’s rank correlation coefficient was determined to evaluate the relationship between continuous variables. Specifically, we evaluated whether there was a correlation between (i) thrombus weight and apixaban peak plasma level and (ii) thrombus weight and daily apixaban dose normalized for preoperative weight. For the intermediary group, the average daily dose over the entire study period was 14 mg (10 mg daily for 6 weeks and 20 mg daily for 4 weeks, thus on average 14 mg daily). Data analysis was performed using Microsoft Office Excel 2016 (Microsoft, USA) and SPSS Statistics (IBM, USA).

## RESULTS

### Perioperative outcomes, survival and apixaban regimen

All 9 pigs underwent uncomplicated surgery and recovered well. Apixaban was commenced at 1 week postoperatively in all animals. One animal in the intermediary-dose group was sacrificed at 9 weeks postoperatively for severe pneumonia. This animal was not excluded because it recovered well initially and because of the chronic aspect of thrombosis seen on valves, as described below. There were no bleeding events.

There was a significant difference in the distribution of preoperative weights between the 3 groups (*H* = 7.0, *P* = 0.03, df = 2), with animals in the intermediary-dose group weighing significantly less than in the high-dose group (*P* = 0.026) (Table 1). When comparing the daily apixaban dose normalized by preoperative weight across the 3 groups, there was a significant difference (*H* = 7.0, *P* = 0.03, df = 2). Animals in the high-dose group received a significantly higher normalized apixaban dose than in the low-dose group (*P* = 0.028) while there was no significant difference between other groups. Table 1 shows an overview of all animals with apixaban regimen, plasma values and thrombus weight on the explanted valve.

### Fluoroscopic evaluation

In 4 animals (animals 1 and 2 from the low-dose group and animals 3 and 4 from the intermediary-dose group, Table 1), fluoroscopic evaluation of cusp motion was clearly disturbed, with either 1 or both cusps completely blocked or showing only minimal motion. The 5 remaining animals from the intermediary and high-dose group showed complete excursion of both cusps, suggesting normal mechanical valve function.

### Macroscopic valve assessment

**Figure 1:**
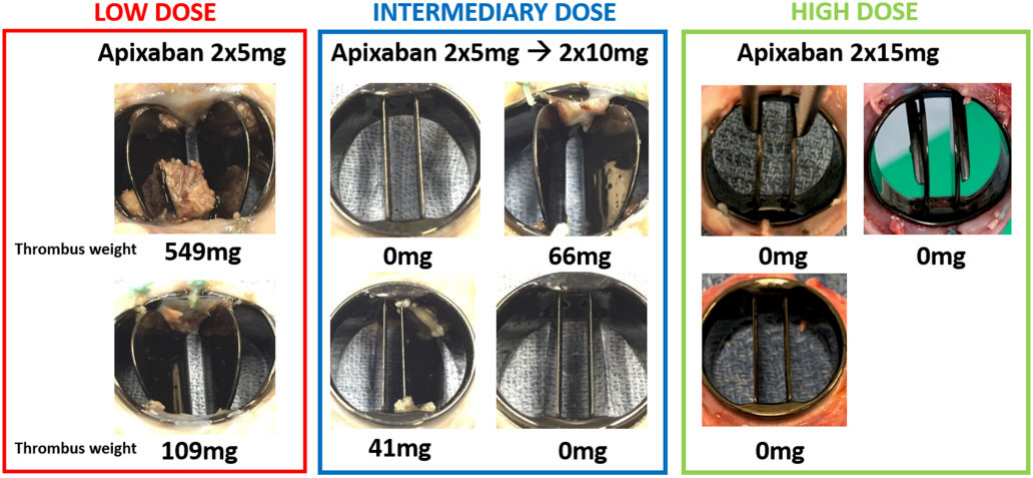
Valve macroscopy and thrombus weight on the explanted valves.

A full necropsy was performed in all animals. There were no macroscopic signs of pulmonary embolism nor were there any macroscopic signs of bleeding complications. Valves in the low-dose group (*n* = 2) showed manifest, chronic thrombosis with 1 and both hinge mechanisms blocked, respectively. One valve was obstructed by a large lesion which was sent for histopathology, confirming an old, fibrotically organized thrombus without signs of endocarditis. In the intermediary-dose group (*n* = 4), the valve appeared clean with no thrombosis and normal mobility of both leaflets in 2/4 animals while 2 other valves showed moderate thrombosis. In the 3 animals receiving 15 mg apixaban twice daily, the valve was clean without thrombosis. An overview of valve macroscopy with thrombus weight for all valves is shown in Fig. 1.

**Figure 2:**
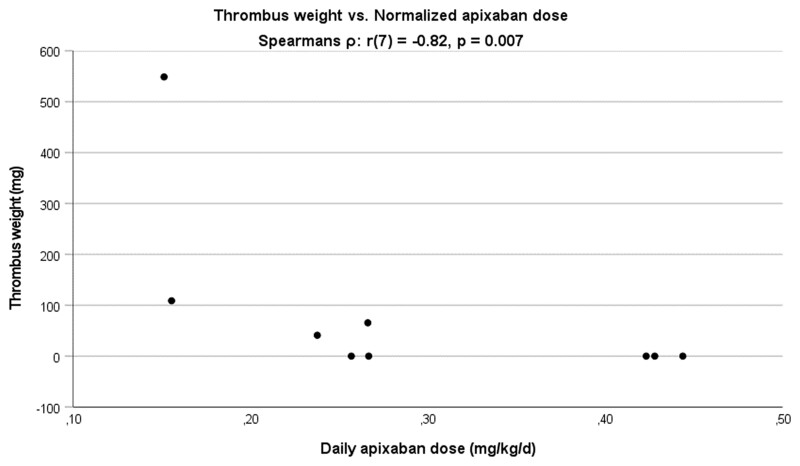
Thrombus weight for each valve plotted against the total daily apixaban dosage (mg/day) normalized for preoperative weight (kg). From left to right, 3 clusters are observed corresponding to the low- (*n* = 2), intermediary- (*n* = 4) and high-dose (*n* = 3) groups. Spearmans’s rank correlation coefficient indicates a negative correlation between apixaban dose and thrombus weight [*r*(7) = −0.82, *P* = 0.007].

There was no significant difference in thrombus weight between the 3 groups (*H* = 5.88, *P* = 0.053, df = 2). Figure 2 shows the thrombus weight on each valve plotted against the daily apixaban dosage (mg/day) normalized for preoperative weight (kg). Spearman’s rank correlation coefficient indicates that there is a negative correlation between apixaban dose and thrombus weight [*r*(7) = −0.82, *P* = 0.007]. There was no correlation between apixaban peak plasma level and thrombus weight [*r*(6) = −0.25, *P* = 0.56].

### Apixaban plasma concentrations

Median apixaban peak plasma concentration prior to sacrifice was 43.6 ng/ml (range 23.1–64), 49.9 ng/ml (range 39.9–61) and 49.9 ng/ml (range 44.6–82.9) for the low-, intermediary- and high-dose groups, respectively (*H* = 0.22, *P* = 0.89, df = 2).

## DISCUSSION

There is an unmet need for alternatives to warfarin in the setting of mechanical heart valves as this remains the most challenging indication for long-term anticoagulation [23]. Safer anticoagulant therapy will improve survival and quality-of-life. Furthermore, the need for chronic anticoagulation is currently steering young adults requiring aortic valve replacement towards the use of tissue valves with still uncertain durability [24]. Because newest generation mechanical valves such as the On-X^®^ aortic valve are approved for reduced INR regimes, they represent part of the solution [4]. DOACs such as apixaban are promising candidates to further reduce thrombo-embolic and bleeding complications [7]. An important obstacle in the evaluation of novel therapeutic strategies is the lack of clinically representative animal models to study mechanisms of valve thrombosis. In this pilot study, we aimed to evaluate the effect of apixaban in a porcine model of mechanical valve thrombosis with newest generation bileaflet mechanical valves implanted in pulmonary position.

We observed manifest valve thrombosis in 2 pigs receiving 5 mg apixaban twice daily. This strongly suggests that pulmonary position in pigs provides an aggressive model for mechanical valve thrombosis, likely due to the aorta-like laminar and pulsatile flow pattern, yet with a lower peak flow velocity and lower transvalvular gradient [25]. Therefore, this model is clinically relevant, as opposed to previous porcine models with heterotopic implantation of mechanical valved conduits bypassing the ligated descending aorta [8, 9, 19, 20]. Most importantly, we show that apixaban has the potential to prevent mechanical valve thrombosis in On-X^®^ valves, even in highly thrombogenic conditions. Additional animals undergoing mechanical valve implantation in pulmonary position without chronic anticoagulant therapy are needed to validate this model.

For patients taking apixaban 5 mg twice daily, peak plasma concentrations range between 128–488 ng/ml [26, 27]. The low plasma concentrations measured in our study, even in animals taking 15 mg twice daily, suggest that oral absorption is limited or that pigs metabolize apixaban rapidly. This is confirmed by a study in which oral administration of very high apixaban dosages (1 mg/kg/d) in pigs led to peak apixaban concentrations of 185 ng/ml [8]. Furthermore, it has been reported that the pig’s response to anticoagulant therapy can vary strongly, as it does in humans [28]. Dose finding studies will determine the ideal and clinically representative dosage for future experiments. Next to merit investigation are molecules acting on the contact activation pathway. Factor XI inhibitors, for example have been shown to prevent venous thrombosis and are expected to be safer than factor X or factor II inhibitors [29].

###  

There are several important limitations to our study. We observed a dosage-dependent effect of apixaban dose on reduction of thrombus weight, yet there was no statistically significant difference in thrombus weight between treatment groups. This is likely due to our small sample size with only 2 animals in the low-dose group. While a dose adjustment during the study in 4 animals makes it challenging to interpret our results, the most important finding remains that apixaban is able to prevent mechanical valve thrombosis in pigs. We evaluated the isolated effect of apixaban on valve thrombosis in an aggressive model, yet patients with an On-X^®^ aortic valve may also receive aspirin in addition to vitamin K antagonists, especially in the setting of a reduced target INR [4]. In this pilot study, we only measured peak apixaban plasma concentrations prior to animal sacrifice. To provide a more comprehensive picture of the effect of apixaban on the porcine coagulation system and its metabolization, future studies should include serial apixaban measurements during follow-up and coagulation assays. Additional experiments in pigs not receiving anticoagulation will enable investigation of the underlying coagulation pathways of valve thrombosis in this model. A complete haemodynamic evaluation in non-operated pigs would enable the comparison of the thrombogenicity of pulmonary and aortic position.

## CONCLUSION

Apixaban has the potential to prevent mechanical valve thrombosis in On-X^®^ valves, even in a highly thrombogenic situation. Further pharmacokinetic studies are needed to determine the ideal apixaban dosage for future porcine experiments and to extrapolate observations to the clinical situation.

## Funding

Lucas Van Hoof is supported by a Doctoral Grant Strategic Basic Research (SB 1S70220N) from the Research Foundation Flanders (FWO). The On-X^®^ aortic valves used in the described experiments were provided by CryoLife (Austin, TX, USA).

**Conflict of interest:** The On-X^®^ aortic valves used in the described experiments were provided by CryoLife (USA). Study design, conduction of the experiments and analyses were performed independently. None of the authors have a conflict of interest to declare.

## Data Availability Statement

Data underlying this article will be shared upon reasonable request to the corresponding author.

## Author contributions

**Lucas Van Hoof:** Data curation; Formal analysis; Investigation; Methodology; Project administration; Visualization; Writing—original draft; Writing—review & editing. **Isabelle Truyers:** Data curation; Formal analysis; Investigation; Methodology; Supervision; Writing—original draft; Writing—review & editing. **Hadewych Van Hauwermeiren:** Data curation; Formal analysis; Investigation; Methodology; Project administration; Validation; Visualization; Writing—original draft; Writing—review & editing. **Bram Nachtergaele:** Data curation; Formal analysis; Investigation; Resources; Visualization; Writing—original draft. **Tom Langenaeken:** Formal analysis; Writing—original draft; Writing—review & editing. **Marc Jacquemin:** Formal analysis; Methodology; Supervision; Writing—original draft. **Filip Rega:** Supervision; Visualization; Writing—original draft; Writing—review & editing. **Peter Verhamme:** Conceptualization; Data curation; Investigation; Methodology; Supervision; Writing—original draft; Writing—review & editing. **Bart Meuris:** Conceptualization; Data curation; Formal analysis; Investigation; Methodology; Project administration; Resources; Supervision; Validation; Visualization; Writing—original draft; Writing—review & editing.

## Reviewer information

Interactive CardioVascular and Thoracic Surgery thanks Leila Louise Benhassen, Elisabet Berastegui, Andrea Colli and the other, anonymous reviewer(s) for their contribution to the peer review process of this article.

## Notes

Presented at the 35th Annual Meeting of the European Association for Cardio-Thoracic Surgery, Barcelona, Spain, 13–16 October 2021.

## ABBREVIATIONS

DOACs: Direct oral anticoagulants
INR: International normalized ratio
